# Unbiased Identification of Dengue Virus Non-Structural Protein 1 Peptides for Use in Vaccine Design

**DOI:** 10.3390/vaccines10122028

**Published:** 2022-11-27

**Authors:** Nikole L. Warner, Susan B. Core, Kathryn M. Frietze

**Affiliations:** 1 Department of Molecular Genetics and Microbiology, University of New Mexico Health Sciences, Albuquerque, NM 87131, USA; 2 Clinical and Translational Science Center, University of New Mexico Health Sciences, Albuquerque, NM 87131, USA

**Keywords:** dengue virus, virus-like particles, bacteriophage, non-structural protein 1, NS1

## Abstract

Dengue virus (DENV) is a global health problem, with over half of the world’s population at risk for infection. Despite this, there is only one licensed vaccine available to prevent infection and safety concerns limit immunization to only a subset of individuals. Most dengue virus vaccine efforts attempt to evoke broadly neutralizing antibodies against structural proteins. However, eliciting antibodies to block the activity of viral proteins involved in pathogenesis could be a useful complementary approach. Studies suggest that non-structural protein 1, which participates in disruption of the endothelial barrier and is hypothesized to play a significant role in the progression to severe dengue, could be a promising target for vaccine efforts. Here, we used an unbiased approach to identify peptide epitopes of dengue virus non-structural protein 1 that could evoke antibodies that bind to NS1 from all 4 serotypes and also bind to DENV-infected cells. DENV-2 NS1 peptides were generated such that 35 overlapping 15 amino acid peptides represented the entire NS1 protein. These peptides were each chemically conjugated to bacteriophage virus-like particles (VLP) and used to immunize mice. Sera were then screened for IgG to cognate peptide as well as binding to recombinant hexameric NS1 from all four DENV serotypes as well as binding to DENV-2 infected cells by microscopy. From these data, we identified several peptides that were able to elicit antibodies that could bind to infected cells as well as DENV NS1. These peptides and their homologues in the corresponding NS1 of other DENV serotypes could be used as potential immunogens to elicit binding antibodies to NS1. Future studies will investigate the functional and protective capacities of antibodies elicited by these immunogens against DENV NS1.

## 1. Introduction

Dengue virus (DENV) is a global health problem, with over half of the world’s population at risk for infection. DENV includes four serotypes (DENV-1, -2, -3, -4) and is primarily transmitted by the *Aedes aegypti* mosquito in tropical and subtropical regions of the world [1]. Infection outcomes can range from asymptomatic, dengue fever, and severe dengue, including dengue hemorrhagic fever and dengue shock syndrome. Severe Dengue (SD) has a high mortality rate, particularly without prompt and appropriate medical care. The risk of SD is increased when an individual experiences a secondary DENV infection, which is when a person who had previously been infected with DENV is infected at a later date with a heterologous serotype of DENV [2]. The increased risk of SD upon secondary infection is thought to be caused by a phenomenon known as Antibody Dependent Enhancement (ADE). This is when pre-existing antibodies to one DENV serotype help facilitate uptake of a heterologous DENV serotype into Fcγ receptor-bearing immune cells, resulting in higher viremia and subsequent increased pathology [2]. The antibodies that facilitate ADE bind to serotype-specific epitopes on the envelope protein of the DENV virion, which is exposed on the surface of the virion. These serotype-specific antibodies can neutralize their cognate DENV serotype. However, these serotype-specific antibodies can bind to heterologous serotypes of DENV but are not neutralizing, allowing for the uptake into Fcγ receptor-bearing immune cells and subsequent enhanced viral production leading to worse disease. Because of the ADE phenomenon, vaccines against DENV must avoid eliciting these ADE promoting antibodies. 

Vascular leakage leading to hemorrhage and shock is a key pathogenic feature of SD. The DENV non-structural protein NS1 is implicated in SD manifestations [3]. As a non-structural protein, NS1 is not a component of the DENV virion, but rather is a viral protein produced in infected cells that is involved in other aspects of virus–host interactions. NS1 is produced early during infection before viral replication occurs and is secreted in large quantities from infected cells into the blood [4]. The detection of high serum levels of soluble NS1 (sNS1) (>600 ng/mL) within the first 72 h of illness is associated with Dengue Hemorrhagic Fever [5]. NS1 is thought to cause plasma leakage by disrupting vascular endothelial cell tight junctions by two proposed mechanisms: (1) NS1 binds to Toll-like receptors (TLRs) on PBMCs, stimulating the production of cytokines (IL-10, IL-6, and TNF-α) that damage the vascular endothelial cell integrity, and (2) NS1 directly binds to vascular endothelial cells and causes the disruption of tight junctions [4,6,7]. The research supporting this comes from cell-culture models of infection and NS1 activity [7,8,9,10,11,12,13], animal models of NS1 activity [7,14,15,16,17,18], and human studies investigating the association of NS1 with clinical progression to SD [5,19]. Taken together, these data provide evidence that excessive NS1 production may drive plasma leakage. For this reason, NS1 has become a promising target for vaccine efforts. 

NS1 vaccines would protect against NS1-mediated pathogenesis but would not be expected to provide sterilizing immunity against viral infection. Additionally, because NS1 is not a structural protein of the DENV virion, antibodies to NS1 would not be capable of causing ADE of infection. A variety of approaches have been used to develop vaccines against DENV NS1. For a review of current vaccine efforts for DENV NS1, please see [20]. Despite the promise of NS1 vaccines for preventing DENV pathogenesis, efforts are complicated by data showing that a subset of antibodies against NS1 can themselves cause damage to endothelial cells through cross-reactivity to host proteins on the endothelial cells and platelets [21,22,23,24,25,26,27]. Although the functional activity of these cross-reactive antibodies in humans has not yet been shown, there are sufficient in vitro and in vivo animal studies to justify caution [18,22,26,27]. Because of this, vaccines against DENV NS1 protein need to elicit antibodies that block NS1 activity but do not cross-react with host proteins. One of the immunodominant epitopes of NS1 in humans and mice is the wing-domain, a disordered region of the protein [28,29]. Importantly, the wing-domain contains a cross-reactive motif, KXWG [26], and antibodies to the wing-domain cross-react with host proteins [18,21,26]. There are additional cross-reactive motifs that have been identified, including ELK/KLE motifs [18]. Additionally, the C-terminus of the NS1 protein has been identified as containing epitopes the elicit cross-reactive antibodies to platelets [25,27]. These epitopes should be avoided in NS1 vaccine antigens for safety reasons.

We previously reported our attempt to identify epitope-specific vaccines against DENV NS1 by targeting conserved regions of NS1, with the hypothesis that these regions may be important for DENV NS1 structure and function, and therefore promising targets for vaccine-elicited antibodies [30]. From those studies, we identified one region that was able to elicit antibodies that could bind to NS1 from all four DENV serotypes. Unfortunately, this conserved epitope corresponded to the cross-reactive wing-domain mentioned above. 

For this reason, we decided to use an unbiased approach to screen overlapping 15-amino acid peptides corresponding to the DENV NS1 protein. We hypothesized that an unbiased approach to peptide identification could reveal new peptide epitopes to target for DENV NS1 vaccination. These peptides were displayed on Qβ virus-like particles (VLPs) and used to immunize mice, allowing us to then characterize the binding capability of the elicited antibodies and identify promising targets for further exploration.

## 2. Materials and Methods

### 2.1. Synthesis of NS1 Peptides

The DENV-2 NS1 protein (Accession #: NP_739584.2) was used to identify overlapping 15 amino acid peptides for investigation. Peptides were custom synthesized by GenScript USA Inc. and confirmed with mass spectrometry. A C-terminal (Gly)_3_-Cys linker was also synthesized on each peptide of interest to allow for chemical conjugation to the surface of Qβ VLPs. 

### 2.2. Qβ VLP Vaccine Production

Qβ VLPs were produced as previously described [30]. Briefly, electrocompetent *Escherichia coli* (Sigma Aldrich) was transformed with a plasmid containing the amino acid sequence for the Qβ coat protein under a lac inducible promoter. Bacteria cultures were grown in LB until an optical density (OD) of 600 was reached, after which cultures were then induced with 0.5 mM Isopropyl-β-D-thiogalactoside (IPTG). Three hours after induction, bacteria were pelleted, and supernatant removed. Pellets were sonicated in the presence of 10% deoxycholate (DOC) to lyse cells and release Qβ VLPs. Sonicated solution was then incubated at 37 °C for 1 h in a final concentration of 2 mM magnesium chloride (MgCl2) and 10 mg/mL of DNase. Samples were centrifuged and supernatants were collected. Ammonium sulfate was added to 60% saturation and kept overnight at 4 °C. Precipitated VLPs were then collected by centrifugation at 12,298× *g* for 10 min. Qβ samples were fractionated by size-exclusion chromatography using Sepharose CL-4B resin (Sigma-Aldrich). Fractions containing VLPs were identified by agarose gel electrophoresis and SDS-PAGE and then precipitated by ammonium sulfate (70% saturation) overnight. Precipitated VLPs were then collected by centrifugation at 12,298× *g* for 10 min. VLPs were resuspended in PBS and then dialyzed against PBS to remove remaining ammonium sulfate. Qβ VLP stocks were then LPS depleted by five sequential Triton-X 100 extraction and concentration was determined by SDS-PAGE gel. Stocks were stored at −20 °C until used. 

To engineer immunogens, synthetic NS1 peptides were then conjugated to the surface of Qβ VLPs through the use of succinimidyl-6-((b-maleimidopropionamido) hexanoate (SMPH). SMPH is a bifunctional crosslinker that binds the surface exposed lysines on Qβ VLPs and bind the C-terminal cysteine containing a free sulfhydryl group. Qβ VLPs allowed to react with SMPH for two hours with rocking at room temperature and excess SMPH was removed by centrifugation using Amicon Ultracel 100K filtration devices (Merck). Peptides were then added at 10ug concentration and incubated at 4 °C overnight. Excess peptide was removed through centrifugation with Amicon Ultracel 100k filtration devices. Conjugation efficiency and concentration of vaccines were estimated by SDS-PAGE electrophoresis and Coomassie staining.

### 2.3. Enzyme-Linked Immunosorbent Assays (ELISAs)

Peptide ELISAs were completed as previously described [30]. Briefly, 96-well ELISA plates were coated with 0.5 μg/50 μL of Streptavidin as a protein carrier (Invitrogen Cat #434302) in phosphate buffered saline (PBS) overnight at 4 °C. Wells were washed three times with PBS and 1 μg/50 μL of succinimidyl-6-((b-maleimidopropionamido) hexanoate (SMPH; Millipore Sigma) in PBS was added to each well for 1 h at room temperature with rocking. Plates were washed three times with PBS and cognate peptide was diluted in PBS and added to each well at a concentration of 1 μg/50 μL for two hours at room temperature with rocking. Plates were then washed three time with PBS and blocked with 150 μL of 0.5% Milk/PBS overnight in 4 °C. Sera samples were then diluted in 0.5% Milk/PBS starting with 1:40 dilution, followed by 4-fold dilutions up to 1:655,360. Plates were washed twice with PBS and sera were plated at 50 μL volumes for two hours at room temperature with shaking. Plates were washed five times with PBS and 50 μL of secondary Goat anti-mouse antibody conjugated with horseradish peroxidase (HRP; Jackson ImmunoResearch) was added at a 1:5000 dilution in 0.5% Milk/PBS for 45 min at room temperature with rocking. Wells were washed 5 times with PBS and 50 μL of soluble TMB was added to each well for 15 min with rocking. Enzymatic reaction was quenched using 50 μL of 1% Hydrochloric acid solution. Plates were analyzed at 450 nm wavelength with an accuSkan plate reader (ThermoFisher). 

For ELISAs testing the binding of antibodies to DENV NS1, soluble NS1 proteins were produced in HEK 293 cells and purchased from The Native Antigen Company. ELISA plates were coated with 0.16 μg/well of NS1 in PBS and incubated overnight at 4 °C. Plates were washed 3 times with PBS and wells were blocked with 100 μL of 0.5% Milk/PBS for one hour at room temperature with rocking. The remaining steps of the ELISA followed the protocol as above. 

### 2.4. Animals

Male and female, 6–8-week-old BALB/c mice (n = 2 or n = 6 per group) were immunized intramuscularly in the hind leg twice, at three-week intervals with 5 μg of vaccine in 50 μL volumes. At day 21, blood samples were collected via retro-orbital bleeds to analyze sera antibodies via peptide ELISA. Day 42, animals were sacrificed via cardiac puncture to collect sera. Mice were purchased from Jackson Labs.

### 2.5. Cells and Viral Stocks

Human embryonic kidney (HEK-293) cells were purchased through American Type Culture Collection (ATCC, CRL-1573). Cells were kept in complete media containing Eagle’s Minimum Essential Medium (MEM, ATCC) supplemented with 10% fetal bovine serum (FBS). *Aedes albopictus* C6/36 cells were purchased through ATCC (CRL-1660) and grown in complete media containing MEM supplemented with 0.1% gentamycin reagent solution (Gibco), 1% of 100X MEM Non-essential amino acids (Gibco), 1% of 100 × 200 mM L-glutamine (Gibco) and 10% FBS.

DENV type 2 (NGC Proto was kindly provided by Dr. Kathryn Hanley at New Mexico State University (NMSU). Viral stocks were propagated in C6/36 cells and stored at −80 °C in C6/36 media supplemented with 1X sucrose phosphate glutamate (SPG) buffer. Viral stocks were verified through sequencing RT-PCR product.

### 2.6. Anti-NS1 Antiserum Binding to DENV-Infected HEK-293 Cells

HEK-293 cells were plated at 25,000 cells/well on Cellvis 96-well glass bottom chimney plates overnight. Cells were then washed once with HEK media and infected DENV-2 NGC Proto at an MOI of 100. Plates were rocked every 5 min for a total of 20 min. After incubation, 150 μL of HEK media was added to each well and plates were incubated for three days at 37 °C. HEK media were removed, and cells were washed once with PBS. Cells were then fixed with 200 μL of 100% methanol per well for 20 min, two times. Fixed cells were washed once with PBS, followed by blocking with 200 μL of 2% goat sera for one hour. Goat sera were removed, and cells were washed three times with PBS. Sera from immunized mice were added at 1:40, 1:80, 1:160 and diluted in 2% goat sera. Commercial antibodies were added at the following dilutions: Mouse anti-Flavivirus NS1 antibody 1:500 (Abcam, ab214337), rabbit anti-flavivirus envelope protein antibody 1:1000 (4G2; The Native Antigen Company). Plates were washed three times with PBS and secondary antibodies were diluted at 1:200 in 2% goat sera for 45 min. Secondary antibodies used were Alexa Fluor 647-conjugated goat anti-rabbit IgG (Jackson ImmunoResearch) and goat anti-mouse IgG conjugated with Alexa Fluor 488 (Abcam). Wells were washed three times with PBS and 50 μL of 1:2000 dilution of Hoechst (Invitrogen) was added to each well for 10 min. Plates were washed three times with PBS and 150 μL of PBS was added to each well for imaging. This research made use of the Fluorescence Microscopy and Cell Imaging Shared Resources which is partially supported by UNM Comprehensive Cancer Center Support Grant. Plates were imaged on a Zeiss Axio Observer epifluorescence microscope with a Hamamatsu Flash 4.0 camera using Slidebook imaging software.

## 3. Results

### 3.1. Strategy for Unbiased Identification of NS1 Peptide Eptiopes of Interest

**Figure 1:**
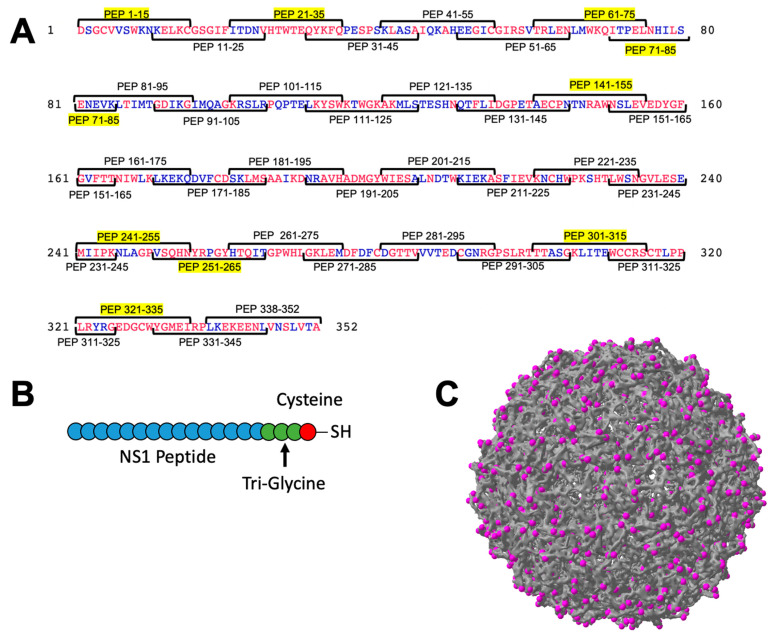
Unbiased design of Qβ VLP vaccines displaying peptide from DENV NS1 protein. The amino acid sequence of DENV-2 was used to identify overlapping peptides of 15 amino acids in length, overlapping by 5 amino acids on each end (**A**). A total of 34 peptides were manufactured and 9 priority peptides are highlighted in yellow (**A**). Conservation of the amino acids among the 4 DENV serotypes is indicated in red (perfectly conserved among all serotypes) or blue (not conserved). Peptides were manufactured with a tri-glycine linker sequence containing a terminal cysteine (**B**) and conjugated to the exposed lysines on the surface of Qβ VLPs (**C**, magenta).

DENV NS1 protein is a 352 amino acid protein detectable in DENV infected cells as well as secreted as a hexameric protein [4,31]. Because the sequence of NS1 is variable among the DENV serotypes, we decided to focus initial efforts on DENV-2. We chose DENV-2 because many of the animal models available for DENV infection use a DENV-2 strain for infection. We chose to synthesize 15 amino acid peptides, overlapping by 5 amino acids, providing us full coverage of the DENV-2 NS1 protein with 35 peptides (Figure 1). These peptides were commercially synthesized with a (Gly)_3_-Cys linker on the C-terminus to allow for chemical conjugation using the free sulfhydryl group provided by the Cys residue. Two of these peptides (Pep 291–305 and Pep 338–352) were not able to be synthesized. Each peptide was conjugated to Qβ VLPs purified from *E. coli*, resulting in 33 individual immunogens for further testing. Four peptides did not successfully conjugate (Pep 81–95, Pep 151–165, Pep 191–205, and Pep 221–235). Conjugation was confirmed using an SDS-PAGE gel with Coomassie staining, demonstrating a shift in electrophoretic mobility indicating conjugation of an estimated 1–3 peptides per individual coat protein (Figure S1).

### 3.2. Immunogenicity of Qβ VLPs Displaying Unbiased NS1 Peptide Epitopes

**Figure 2:**
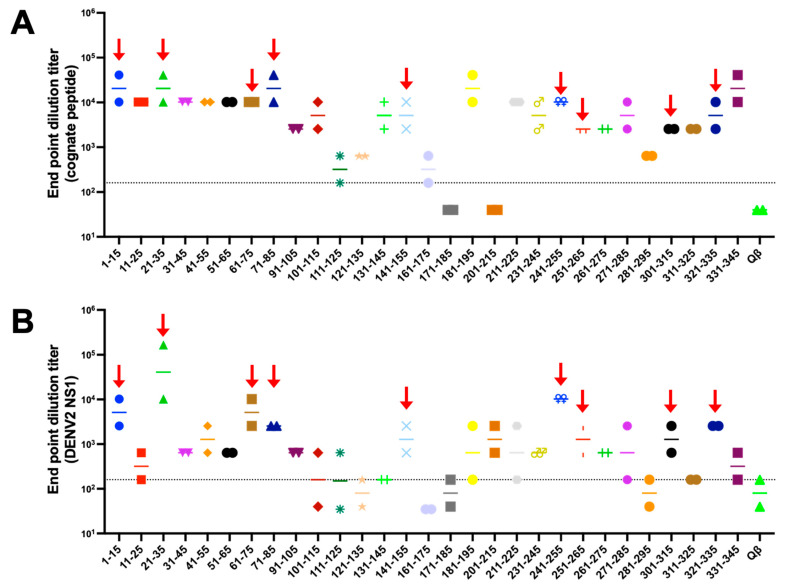
Qβ VLP vaccines displaying DENV NS1 peptide elicit variable antibody responses in mice. BALB/c mice (n = 2) were immunized intramuscularly 2 times with 5 μg of Qβ VLP vaccines and blood was collected 3 weeks after the final immunization. ELISAs against the cognate peptides (**A**) and DENV-2 NS1 (**B**) were carried out and endpoint dilution titer was determined. Red arrows indicate 9 peptides prioritized for further investigation. Each peptide is shown is different colors to help distinguish them in the graph.

Having successfully engineered 32 Qβ VLPs displaying NS1 peptides, we next performed an initial immunogenicity screen of the immunogens in mice. We immunized two mice per group to minimize the number of animals used in this initial screen of immunogens. Mice were immunized twice, 3 weeks apart, and serum was collected 3 weeks after boost. Of the 35 immunogens evaluated, all but two elicited antibodies that bound to cognate peptide above Qβ negative control (Figure 2). Next, we assessed binding of immune sera to recombinant hexameric NS1 from DENV-2 by ELISA (Figure 2). Here, we observe a variety of binding activity among the immunogens, ranging from <1:40 to >1:655,360. In order to prioritize immunogens for further investigation, we established the following criteria: (1) both mice in a group showing endpoint titer higher than 1:160 to cognate peptide, and (2) binding above background to DENV-2 NS1 by ELISA. Using these criteria, we down selected our initial 35 immunogens to nine priority immunogens.

### 3.3. Structural Locations of Down-Selected NS1 Peptide Epitopes

**Figure 3:**
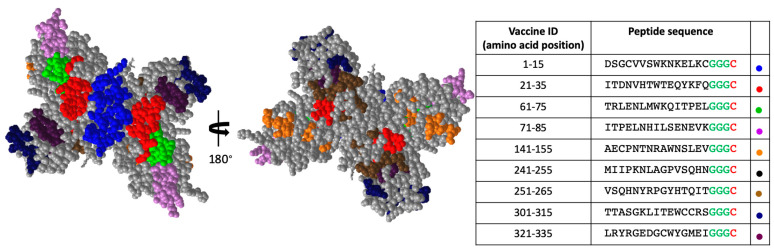
Structural locations of priority NS1 peptides. The 9 peptides identified for additional analysis are shown in various colors. The hexameric internal view of the dimer (**left**) and the hexameric external view of the dimer (**right**) are shown. Glycine linker is shown for each peptide in green and cysteine used for chemical conjugation to VLP is shown in red in table. Colored dots in table indicate the color used in structure for the indicated peptide.

Figure 3 shows the structural locations of each of the peptides we identified in our screen. The sequences of these peptides along with their amino acid position are highlighted in Figure 1. The NS1 protein exists in its soluble form as a hexamer (ref). Figure 3 shows the dimeric form of the NS1 protein. The structure on the left is the view of the NS1 dimer that would be internal to the hexameric form, and the structure on the right is the view of the NS1 dimer that would be external (exposed) in the hexameric form. Some of the peptide we identified in our unbiased screen appear to be only exposed on the inner surface of the hexamer (Pep 61–75, Pep 321–335), while others have at least some exposure on the predicted hexameric structure of NS1. 

### 3.4. Binding Characteristics of IgG Elicited by Qβ VLPs Displaying NS1 Peptides Epitopes

**Figure 4:**
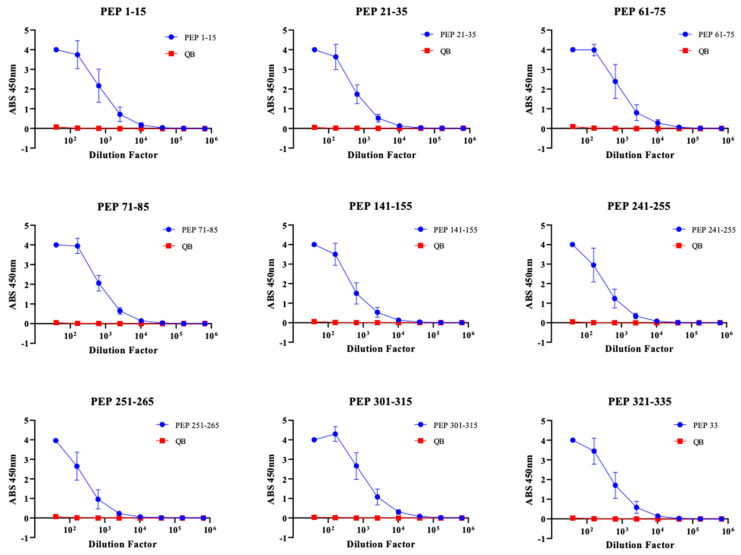
Immunization of BALB/c mice with 9 priority Qβ VLP vaccines induce high titer antibodies against cognate peptide. The top nine Qβ-VLP vaccines identified from the initial screening were used to immunize 6–8-week-old BALB/c mice (n = 6 mice/vaccine, 3 male and 3 female). Peptide ELISA was used to determine IgG binding to cognate peptide comparing vaccine sera (blue) and control sera of mice immunized with Qβ only (red).

**Figure 5:**
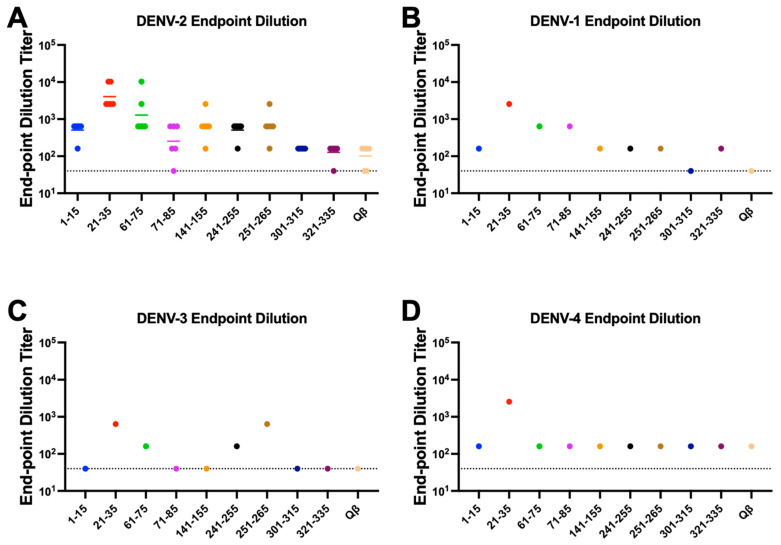
Binding of Qβ-VLP vaccine-elicited antibodies to DENV 1–4 NS1. Mice (n = 6) were immunized 2 times with 5 μg of Qβ-VLP conjugated to peptide and blood was collected 3 weeks after the final immunization. ELISAs against the recombinant hexameric NS1 from the 4 DENV serotypes were used to determine the end-point dilution titer. Individual serum samples were used for DENV-2 NS1 (**A**), and pooled samples were used for DENV-1 (**B**), −3 (**C**), and −4 (**D**) due to limited serum volume available. Colors are used to distinguish each peptide in the graphs.

**Figure 6:**
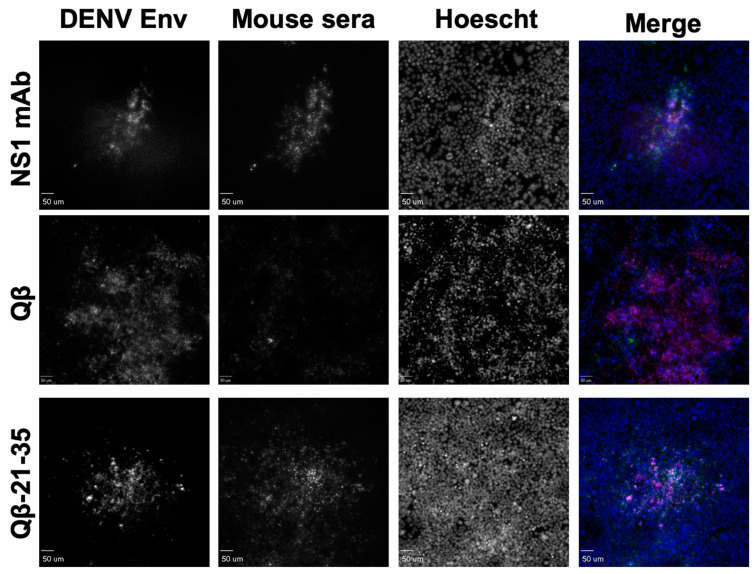
Binding of Qβ-VLP-21–35 vaccine-elicited antibodies to DENV-2 infected cells. Mice (n = 6) were immunized 2 times with 5 μg of Qβ-VLP-21–35 and blood was collected 3 weeks after the final immunization. Cells infected with DENV-2 were fixed and stained with sera from mice immunized with Qβ-VLP-21–35, Qβ alone (negative control), or a commercially available mAb to NS1 (green in merge). Cells were then imaged by confocal microscopy and co-localization of DENV envelope (red in merge), and mouse sera were determined.

Having down selected our immunogens to 9, we next repeated our immunizations with larger groups (n = 6, male and female) to obtain serum volumes sufficient to perform additional binding experiments. Again, we observed consistently high titer antibody responses to these immunogens after two immunizations without exogenous adjuvant, indicating the high immunogenicity of these immunogens (Figure 4). DENV NS1 exists as a cell-associated dimer as well as a soluble, secreted hexamer [32]. In order to define the binding capability of serum IgG elicited by our immunogens, we performed an ELISA against recombinant hexameric NS1 from all four DENV serotypes as well as immunofluorescence microscopy against DENV-2 infected cells. Serum IgG from immunized mice exhibited binding to DENV-2 NS1 above Qβ control serum for seven of our nine down-selected peptides. However, we observed variable binding of immune sera to NS1 from the other DENV serotypes, with only Peptide 21–35 showing binding to NS1 from all 4 serovars (Figure 5). Immunofluorescence microscopy using immune sera from immunized mice showed that serum IgG from five of the nine immunogens could bind to DENV-2 infected cells (i.e., cell-associated NS1), including Peptide 21–35 (Figure 6, Figure S2).

## 4. Discussion

Here, we report our efforts to conduct an unbiased screen of DENV NS1 peptides to identify potential immunogens for a DENV NS1 vaccine. Overlapping 15 amino acid peptides from DENV-2 NS1 were synthesized and chemically conjugated to the highly immunogenic Qβ virus-like particle platform. These were then screened for their ability to elicit antibodies that bound to cognate peptide and DENV-2 NS1. The 35 original peptides were down selected to 9. Of these, one peptide, corresponding to amino acids 21–35 of DENV-2 NS1, was able to elicit antibodies that bound to NS1 from all four DENV serotypes and also to DENV-2 infected cells. Peptide 21–35 is in a highly conserved region of DENV NS1. 

We previously reported the investigation of the immunogenicity of conserved regions of DENV NS1 protein [30]. Using a biased approach focused on conserved amino acid regions only, we were only able to identify one peptide (amino acids 112–122) that could elicit antibodies that bound to all four DENV serotypes. This is despite only choosing peptides that were perfectly conserved among the DENV serotypes. Our unbiased screen included Peptide 111–125, which was not identified in our top peptides of interest for down selection. Peptide 112–122 from our previous work elicited antibodies that bound to NS1 from all four DENV serotypes but could be a poor choice for vaccination since antibodies to this region have been reported to be cross-reactive with host proteins and may contribute to auto-immune type responses [16,18,21,23,26,27]. Additionally, one of the peptides identified previously encompassed amino acids 25–35, which is perfectly conserved among the DENV serotypes. However, in contrast to Peptide 21–35 identified here, Peptide 25–35 was unable to elicit antibodies that could bind to hexameric NS1 despite eliciting high titer antibodies to the cognate peptide. This highlights the power of performing an unbiased screen such as described here and suggests that targeting only the conserved amino acids may not be sufficient in some cases to elicit antibodies that bind to their antigen in native conformation. Additionally, the addition of four amino acids may have increased the binding ability of the elicited antibodies. 

According to current models of NS1 dimer and hexamer structure, peptide 21–35 is primarily unexposed and unlikely to be accessible to antibodies. That is particularly intriguing considering that IgG from mice immunized against Peptide 21–35 was capable of binding to both hexameric and cell-associated (dimeric) NS1. It is possible that the structural models of NS1 are not accurate for these amino acids and that they are indeed exposed and available for antibody binding, there may be transient exposure of these amino acids that allow antibody binding, or our experimental conditions (fixative or incubation times) could have allowed for the exposure of these amino acids. We also cannot rule out that the soluble hexameric NS1 we used for our ELISAs contained some dimeric NS1 as well. In screens of sera from mice and humans infected with DENV, this peptide was not identified as eliciting antibody responses. Future studies will investigate whether vaccine-induced antibodies to this peptide can block NS1 activity in vitro and also the ability of this immunogen to prevent vascular leak and pathology in an animal model of DENV vascular leak. 

## Data Availability

The data presented in this study are available in the article.

## Supplementary Materials

The following are available online at https://www.mdpi.com/article/10.3390/vaccines10122028/s1, Figure S1: SDS-PAGE. Figure S2: Qβ-VLP vaccine elicited IgG binding to DENV2 infected cells.

## References

[1] Bhatt S., Gething P.W., Brady O.J., Messina J.P., Farlow A.W., Moyes C.L., Drake J.M., Brownstein J.S., Hoen A.G., Sankoh O. (2013). The Global Distribution and Burden of Dengue. Nature.

[2] Halstead S.B. (2014). Dengue Antibody-Dependent Enhancement: Knowns and Unknowns. Microbiol. Spectr..

[3] Malavige G.N., Ogg G.S. (2017). Pathogenesis of Vascular Leak in Dengue Virus Infection. Immunology.

[4] Chen H.R., Lai Y.C., Yeh T.M. (2018). Dengue Virus Non-Structural Protein 1: A Pathogenic Factor, Therapeutic Target, and Vaccine Candidate. J. Biomed. Sci..

[5] Libraty D.H., Young P.R., Pickering D., Endy T.P., Kalayanarooj S., Green S., Vaughn D.W., Nisalak A., Ennis F.A., Rothman A.L. (2002). High Circulating Levels of the Dengue Virus Nonstructural Protein NS1 Early in Dengue Illness Correlate with the Development of Dengue Hemorrhagic Fever. J. Infect. Dis..

[6] Puerta-Guardo H., Glasner D.R., Harris E. (2016). Dengue Virus NS1 Disrupts the Endothelial Glycocalyx, Leading to Hyperpermeability. PLoS Pathog..

[7] Glasner D.R., Ratnasiri K., Puerta-Guardo H., Espinosa D.A., Beatty P.R., Harris E. (2017). Dengue Virus NS1 Cytokine-Independent Vascular Leak Is Dependent on Endothelial Glycocalyx Components. PLoS Pathog..

[8] Chen H.R., Chao C.H., Liu C.C., Ho T.S., Tsai H.P., Perng G.C., Lin Y.S., Wang J.R., Yeh T.M. (2018). Macrophage Migration Inhibitory Factor Is Critical for Dengue NS1-Induced Endothelial Glycocalyx Degradation and Hyperpermeability. PLoS Pathog..

[9] Modhiran N., Watterson D., Muller D.A., Panetta A.K., Sester D.P., Liu L., Hume D.A., Stacey K.J., Young P.R. (2015). Dengue Virus NS1 Protein Activates Cells via Toll-like Receptor 4 and Disrupts Endothelial Cell Monolayer Integrity. Sci. Transl. Med..

[10] Modhiran N., Watterson D., Blumenthal A., Baxter A.G., Young P.R., Stacey K.J. (2017). Dengue Virus NS1 Protein Activates Immune Cells via TLR4 but Not TLR2 or TLR6. Immunol. Cell Biol..

[11] Chen J., Ng M.M., Chu J.J. (2015). Activation of TLR2 and TLR6 by Dengue NS1 Protein and Its Implications in the Immunopathogenesis of Dengue Virus Infection. PLoS Pathog..

[12] Beatty P.R., Puerta-Guardo H., Killingbeck S.S., Glasner D.R., Hopkins K., Harris E. (2015). Dengue Virus NS1 Triggers Endothelial Permeability and Vascular Leak That Is Prevented by NS1 Vaccination. Sci. Transl. Med..

[13] Adikari T.N., Gomes L., Wickramasinghe N., Salimi M., Wijesiriwardana N., Kamaladasa A., Shyamali N.L., Ogg G.S., Malavige G.N. (2016). Dengue NS1 Antigen Contributes to Disease Severity by Inducing Interleukin (IL)-10 by Monocytes. Clin. Exp. Immunol..

[14] Schlesinger J.J., Brandriss M.W., Walsh E.E. (1987). Protection of Mice against Dengue 2 Virus Encephalitis by Immunization with the Dengue 2 Virus Non-Structural Glycoprotein NS1. J. Gen. Virol..

[15] Henchal E.A., Henchal L.S., Schlesinger J.J. (1988). Synergistic Interactions of Anti-NS1 Monoclonal Antibodies Protect Passively Immunized Mice from Lethal Challenge with Dengue 2 Virus. J. Gen. Virol..

[16] Lai Y.C., Chuang Y.C., Liu C.C., Ho T.S., Lin Y.S., Anderson R., Yeh T.M. (2017). Antibodies Against Modified NS1 Wing Domain Peptide Protect Against Dengue Virus Infection. Sci. Rep..

[17] Wan S.W., Lu Y.T., Huang C.H., Lin C.F., Anderson R., Liu H.S., Yeh T.M., Yen Y.T., Wu-Hsieh B.A., Lin Y.S. (2014). Protection against Dengue Virus Infection in Mice by Administration of Antibodies against Modified Nonstructural Protein 1. PLoS ONE.

[18] Falconar A.K. (2007). Antibody Responses Are Generated to Immunodominant ELK/KLE-Type Motifs on the Nonstructural-1 Glycoprotein during Live Dengue Virus Infections in Mice and Humans: Implications for Diagnosis, Pathogenesis, and Vaccine Design. Clin. Vaccine Immunol..

[19] Jayathilaka D., Gomes L., Jeewandara C., Jayarathna G.S.B., Herath D., Perera P.A., Fernando S., Wijewickrama A., Hardman C.S., Ogg G.S. (2018). Role of NS1 Antibodies in the Pathogenesis of Acute Secondary Dengue Infection. Nat. Commun..

[20] Lebeau G., Lagrave A., Ogire E., Grondin L., Seriacaroupin S., Moutoussamy C., Mavingui P., Hoarau J.-J., Roche M., Krejbich-Trotot P. (2021). Viral Toxin NS1 Implication in Dengue Pathogenesis Making It a Pivotal Target in Development of Efficient Vaccine. Vaccines.

[21] Falconar A.K. (1997). The Dengue Virus Nonstructural-1 Protein (NS1) Generates Antibodies to Common Epitopes on Human Blood Clotting, Integrin/Adhesin Proteins and Binds to Human Endothelial Cells: Potential Implications in Haemorrhagic Fever Pathogenesis. Arch. Virol..

[22] Lin Y.S., Yeh T.M., Lin C.F., Wan S.W., Chuang Y.C., Hsu T.K., Liu H.S., Liu C.C., Anderson R., Lei H.Y. (2011). Molecular Mimicry between Virus and Host and Its Implications for Dengue Disease Pathogenesis. Exp. Biol. Med..

[23] Sun D.S., King C.C., Huang H.S., Shih Y.L., Lee C.C., Tsai W.J., Yu C.C., Chang H.H. (2007). Antiplatelet Autoantibodies Elicited by Dengue Virus Non-Structural Protein 1 Cause Thrombocytopenia and Mortality in Mice. J. Thromb. Haemost..

[24] Cheng H.J., Lei H.Y., Lin C.F., Luo Y.H., Wan S.W., Liu H.S., Yeh T.M., Lin Y.S. (2009). Anti-Dengue Virus Nonstructural Protein 1 Antibodies Recognize Protein Disulfide Isomerase on Platelets and Inhibit Platelet Aggregation. Mol. Immunol..

[25] Chen M.C., Lin C.F., Lei H.Y., Lin S.C., Liu H.S., Yeh T.M., Anderson R., Lin Y.S. (2009). Deletion of the C-Terminal Region of Dengue Virus Nonstructural Protein 1 (NS1) Abolishes Anti-NS1-Mediated Platelet Dysfunction and Bleeding Tendency. J. Immunol..

[26] Liu I.J., Chiu C.Y., Chen Y.C., Wu H.C. (2011). Molecular Mimicry of Human Endothelial Cell Antigen by Autoantibodies to Nonstructural Protein 1 of Dengue Virus. J. Biol. Chem..

[27] Cheng H.J., Lin C.F., Lei H.Y., Liu H.S., Yeh T.M., Luo Y.H., Lin Y.S. (2009). Proteomic Analysis of Endothelial Cell Autoantigens Recognized by Anti-Dengue Virus Nonstructural Protein 1 Antibodies. Exp. Biol. Med..

[28] Akey D.L., Brown W.C., Dutta S., Konwerski J., Jose J., Jurkiw T.J., DelProposto J., Ogata C.M., Skiniotis G., Kuhn R.J. (2014). Flavivirus NS1 Structures Reveal Surfaces for Associations with Membranes and the Immune System. Science.

[29] Hertz T., Beatty P.R., MacMillen Z., Killingbeck S.S., Wang C., Harris E. (2017). Antibody Epitopes Identified in Critical Regions of Dengue Virus Nonstructural 1 Protein in Mouse Vaccination and Natural Human Infections. J. Immunol..

[30] Warner N.L., Frietze K.M. (2021). Development of Bacteriophage Virus-Like Particle Vaccines Displaying Conserved Epitopes of Dengue Virus Non-Structural Protein 1. Vaccines.

[31] Glasner D.R., Puerta-Guardo H., Beatty P.R., Harris E. (2018). The Good, the Bad, and the Shocking: The Multiple Roles of Dengue Virus Nonstructural Protein 1 in Protection and Pathogenesis. Annu. Rev. Virol..

[32] Muller D.A., Landsberg M.J., Bletchly C., Rothnagel R., Waddington L., Hankamer B., Young P.R. (2012). Structure of the Dengue Virus Glycoprotein Non-Structural Protein 1 by Electron Microscopy and Single-Particle Analysis. J. Gen. Virol..

