# Supplementary material for: Unbiased Identification of Dengue Virus Non-Structural Protein 1 Peptides for Use in Vaccine Design
Source: Vaccines (Basel). 2022 Nov 27;10(12):2028. doi: 10.3390/vaccines10122028 (PMC9784660; doi:10.3390/vaccines10122028)
Supplement: Supplementary file 1 [file vaccines-10-02028-s001.zip › vaccines-2012886-supplementary.pdf]

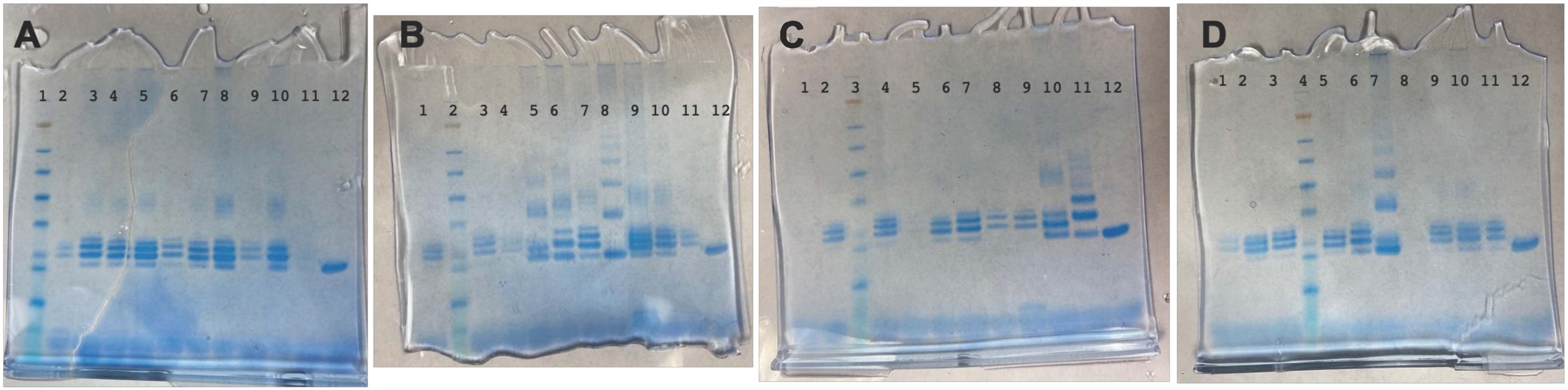

E

	1	2	3	4	5	6	7	8	9	10	11	12
A	Ladder	N/A	1-15	11-25	21-35	31-45	41-55	51-65	61-75	71-85	81-95	Qbeta
B	91-105	Ladder	101-115	111-125	121-135	131-145	141-155	151-165	161-175	171-185	181-195	Qbeta
C	191-205	201-215	Ladder	210-225	221-235	231-245	241-255	251-265	261-275	271-285	281-295	Qbeta
D	291-305	301-315	311-325	Ladder	321-335	331-345	338-352	N/A	N/A	N/A	N/A	Qbeta

Supplemental Figure S1. SDS-PAGE analysis of peptide-VLP conjugations. Full, unaltered images of SDS-PAGE gels stained with Coomassie are presented, showing the conjugation of each peptide to Qbeta VLPs. Identify of each lane is indicated in (**E**). N/A refers to unrelated conjugations that were included in these gels. Lanes are included (empty) for peptides that were unable to be synthesized to simplify the loading strategy. Yellow highlight indicates a peptide that did not conjugate or was not successfully synthesized.

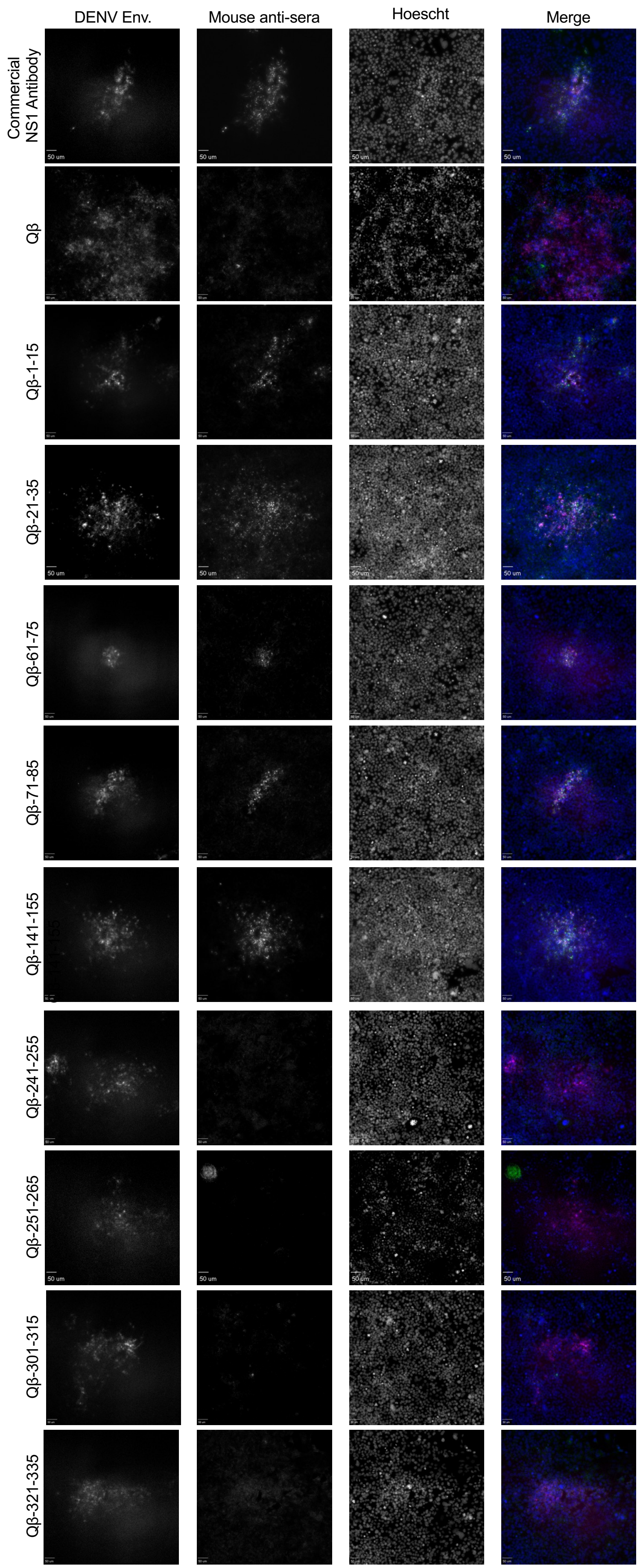

Supplemental Figure S2. Binding of Qβ-VLP-NS1 peptide vaccine-elicited antibodies to DENV-2 infected cells. Mice (n=6) were immunized 2 times with 5ug of vaccine and blood was collected 3 weeks after the final immunization. Cells infected with DENV-2 were fixed and stained with sera from mice immunized with vaccines, Qβ alone (negative control), or a commercially available mAb to NS1 (green in merge). Cells were then imaged by confocal microscopy and co-localization of DENV envelope (red in merge), and mouse sera was determined.
